# Exploring the Association Between Resilience and Quality of Life Among Glaucoma Patients: Sleep Disturbance as a Mediating Factor

**DOI:** 10.3389/fmed.2022.842864

**Published:** 2022-11-09

**Authors:** Qinqi Peng, Bo Qu, Kristin K. Sznajder, Qiongli Chen, Jiahui Fu, Shan He, Xiaoshi Yang

**Affiliations:** ^1^Department of Social Medicine, College of Health Management, China Medical University, Shenyang, China; ^2^Department of Ophthalmology, Fourth Affiliated Hospital of China Medical University, Eye Hospital of China Medical University, Key Laboratory of Lens Research of Liaoning Province, Shenyang, China; ^3^Department of Public Health, Pennsylvania State University College of Medicine, Hershey, PA, United States

**Keywords:** sleep disturbance, resilience, quality of life, glaucoma, structural equation

## Abstract

**Background:**

Patients with glaucoma may experience many symptoms such as blindness, which seriously affect their quality of life (QoL). Resilience is playing a vital role in enhancing the QoL and well-being of patients with chronic diseases. In addition, sleep disturbance is common in patients with glaucoma, leading to a decline in their QoL. However, there is a dearth of research on whether sleep disturbance plays a mediating role between resilience and QoL among glaucoma patients.

**Objective:**

The aim of this study is to explore the role of sleep disturbance in the relationship between resilience and QoL among glaucoma patients.

**Methods:**

From July to December 2019, a cross-sectional survey was conducted on 215 glaucoma patients in an ophthalmic hospital in Liaoning Province. Hierarchical multiple regression (HMR) analyses and structural equation modeling (SEM) were conducted to examine the factors related to QoL and to test the hypothesis that sleep disturbance mediates the relationship between resilience and QoL among glaucoma patients.

**Results:**

The average QoL score among glaucoma patients was 43.85 ± 14.97 as reported by the Glaucoma Quality of Life-15 (GQL-15) scale, where a higher scores indicating a poorer QoL. Resilience was found to be linked with a lower QoL score (*P* < 0.01), while sleep disturbance was associated with a higher QoL score (*P* < 0.01). When sleep disturbance was included in the model as partial mediator, the path coefficients for the association between resilience and QoL score was significantly decreased (a*b = −0.1, BCa95% CI: −0.154∼−0.045).

**Conclusion:**

Findings of this study reflected that QoL among glaucoma patients in China was poor. Resilience was found to be an important positive factor, which could result in the improvement of QoL. Furthermore, sleep disturbance mediated the relationship between resilience and QoL among patients with glaucoma, thereby reducing the positive impact of resilience on QoL in glaucoma patients. Efforts to improve QoL among glaucoma patients may benefit from interventions that enhance the levels of resilience and promote healthy sleep.

## Introduction

Glaucoma is a chronic lifelong disease characterized by concave atrophy of optic papilla and the loss of retinal ganglion cells, which constitutes a major concern for public health (1). According to research statistics, more than 76 million people currently suffer from this disease, with is expected to increase to 112 million by 2040 (2). Because the visual impairment caused by glaucoma is irreversible, the impact on patients is not only physiological, but also psychological. Glaucoma patients may experience psychological problems such as tension, fear, anxiety, pessimism and depression (3), which will affect the recovery of vision, and also the QoL of patients. Therefore, QoL is an important index to evaluate the treatment effect of glaucoma patients (4).

Resilience is the ability to actively adapt to adversity. It can guide individuals to alleviate negative emotions and improved QoL (5). By understanding the broaden-and-build theory of positive emotions, we can realize that positive emotions (such as happiness and interest) broaden people’s thought and action, overcome the physiological effects of negative emotions, enhance resilience, and lead to the rise of emotional well-being, thereby improving individual’s QoL (6, 7). Additionally, the systematic self-reflection model of resilience strengthening shows that those who have experienced hardships, or even trauma, will have greater resilience than those who did not experience (8). Therefore, when glaucoma patients experience adversities such as visual impairment, headaches and eye swelling, these uncomfortable symptoms may stimulate improved resilience, which could increase patients’ treatment compliance, this improving their QoL. Additionally, most studies have suggested that people with high level of resilience had a higher QoL in general (9–12). For example, research by Craig et al. (13) has indicated the QoL of cancer patients who have higher level of resilience was significant better than those with lower level of resilience. Chen et al. (14) also reported that patients with hypertension improved their QoL by focusing actively and strategically on improving their resilience. While the association between resilience and QoL has been studied for many clinical conditions, there is a paucity of research regarding resilience and QoL among glaucoma patients in China.

Sleep disturbance is a common and severe issue among glaucoma patients. According to the previous research, more than 60% of glaucoma patients report having sleep disturbance (15). The occurrence of sleep disturbance among glaucoma patients is considered to be related to visual field damage (16) and circadian rhythm disorder (17). A growing number of studies have documented that through various mechanisms (attention transfer and cognitive change), sleep impacts the generation and regulation of emotion (18, 19). Previously published studies have showed that sleep disturbance is caused by visual field loss and is related to depression and psychological factors (20, 21). Besides, It has been confirmed that the QoL of nocturia patients who reported sleep disturbance appeared to be worse than those without, with sleep disturbance have a greater psychological impact (22). An earlier study on the QoL of pregnant women also stated that stress caused physical and mental responses that affected people’s resilience. In turn, these physiological and psychological outcomes were found to be associated with sleep disturbance (23, 24). Therefore, we hypothesize that sleep disturbance will play a mediating role between resilience and the QoL of glaucoma patients.

Few studies, however, have explored the relationship between resilience and sleep disturbance among Chinese glaucoma patients, and fewer have examined the mediating effect of sleep disturbance in the association between resilience and QoL among glaucoma patients. Therefore, as noted, the aim of this study was to verify the following research hypotheses. Hypothesis 1: resilience is positive factor affecting QoL among glaucoma patients; Hypothesis 2: sleep disturbance is negatively associated with QoL; Hypothesis 3: sleep disturbance mediates the effect of resilience on QoL among glaucoma patients.

## Materials and Methods

### Survey Process and Participants

From July 29 to December 30 in 2019, a cross-sectional, hospital-based investigation was carried out among glaucoma outpatients who met the criteria and be selected continuously in an ophthalmic outpatient hospital in Liaoning Province. The trained investigator conducted face-to-face interviews using a mobile phone enabled questionnaire to help the patients to fill in the questionnaire. Before the questionnaire was conducted, the informed consent of the patients was obtained.

The inclusion criteria for participating were as follows: (1) aged 18 years and above; (2) diagnosed with glaucoma; and (3) agreed to voluntary participation in the survey (25). The exclusion criteria were: (1) glaucoma patients with other systemic diseases such as cancer etc. (25); (2) current diagnosis of substance abuse or addiction; (3) lifetime diagnosis of a psychotic/affective disorder, and (4) prescribed anti-depressants, antipsychotics, or immunosuppressants (17).

### Ethics Considerations

Before the investigation, all participants were fully informed the purpose and relevant contents of this study. The study was conducted based on the Helsinki Declaration revised in 1989, and the study protocols ware also approved by the Ethics Committee of China Medical University.

### Survey Instruments

#### Demographic Characteristics of Glaucoma Patients

Demographic characteristics of glaucoma patients were collected and included: age, gender, marital status, educational, monthly income, duration of the glaucoma disease, duration of other chronic diseases, disease types, number of operations, and family history of glaucoma/cataract. “Age” was group into “≤65 years old” or “>65 years old”; “Marital status” was dichotomized as “married” or “others”; “Educational level” was classified as “Junior high school and below” or “Senior high school and above”; “Monthly income” was divided into “<3000RMB,”“3000-6000RMB,” and “>6000RMB”; “Duration of glaucoma and other chronic disease” was dichotomized as “<3 years” and “≥3 years”; “The disease types” were divided into “Glaucoma” and “Glaucoma complicated with Cataract”; “The number of operations” was “0,” “1,” “2,” “3 or more.”

#### Quality of Life of Glaucoma Patients

The Glaucoma Quality of Life-15 (GQL-15) scale is one of the most effective tools to measure the QoL of glaucoma patients and is often used in research (26–28). There are 15 items in the GQL-15, which measure patients’ peripheral vision, visual acuity, near vision, light and dark vision, glare, and outdoor activity ability. According to the degree of difficulty in completing daily life, the answers to each item are divided into five levels: no difficulty (1 point), minor difficulty (1 point), moderate difficulty (2 points), great difficulty (3 points), and extremely difficulty (4 points). In addition, there is an answer “I can’t complete this daily activity because of my eyes”, which is recorded as 0. The total possible score of the questionnaire was 75. The higher the score, the worse the QoL as measured by the GQL-15. The Cronbach’s alpha coefficient for the scale in this study was 0.966.

#### Sleep Disturbance of Glaucoma Patients

The PROMIS Sleep Disturbance Short Form-8 (Promis8b) (29) was used to evaluate the sleep of study participants over the past 7 days. The scale consists of 8 items, and each item has a 5-point scale: never (1 point), seldom (2 point), sometimes (3), frequently (4 point), everyday (5 point). Items 1, 4, 5 and 6 were scored positively, while items 2, 3, 7 and 8 were reverse scored. The total score of the questionnaire was calculated and possible the score range was 8-40. After t conversion of the original score, it was divided into four levels according to the T-score: no sleep disorder (*t* < 55.0), mild sleep disturbance (55.0-59.9), moderate sleep disturbance (60.0-69.9), and severe disturbance (>70). Higher score indicated more serious sleep disturbance. The Cronbach’s alpha coefficient for the scale in this study was 0.963.

#### Resilience of Glaucoma Patients

The Ego-resiliency scale (ER89) is used to access the levels of resilience among patients. The resilience scale has good reliability and validity and is widely used in China. There are 14 items in the Resilience Scale developed by Block and Kreman (30), which adopts 4-point scoring representing “not applicable at all” to “very applicable”. Higher total scores mean higher levels of resilience. The Cronbach’s alpha coefficient for the scale in this study was 0.845.

### Statistical Analysis

All statistical analyses were performed by using SPSS software IBM version 23.0. *T*-tests and ANOVAs were first applied to evaluate the differences of QoL by demographic and clinical characteristics of glaucoma patients. Secondly, the correlations of sleep disturbance, resilience and QoL were examined by using the Spearman correlation. Next, hierarchical multiple regression (HMR) analysis was used to determine the predictors and mediators related to the QoL of glaucoma patients. Then, structural equation modeling (SEM) was used to asssess the mediating role of sleep disturbance between resilience and QoL in glaucoma patients, which was analyzed by AMOS 17.0. The SEM model included QoL as a dependent variable, resilience as an independent variable and sleep disturbance as a mediator variable. The results were consistent with the SEM criteria (χ2/df <5, GFI >0.90, CFI > 0.90, RMSEA <0.08, and TLI > 0.90). The bootstrap estimate was based on 5000 random samples (a*b products) obtained from the original data, which was used to examine the mediating effect of sleep disturbance between resilience and QoL. The bias-corrected and accelerated 95% CI of each product was investigated. Statistical tests were considered significant if *P* < 0.05 (two-tailed).

## Results

### Description of Demographic of Glaucoma Patients

Table 1 shows the demographic characteristics of glaucoma patients in this study, with an average age of 66.24 ± 12.53 years. Among the 215 patients involved in the survey, 126 (58.6%) were 65 years old and over, 117 (54.4%) were women, and the majority of glaucoma patients (94.4%) were married. In terms of educational level, 59.6% of glaucoma patients reported their highest level of educational junior high school or below. Nearly one fifth of patients (45.6%) reported a monthly income of 3000–6000 RMB, and only 18.6% of glaucoma patients have reported a monthly income of more than 6000 RMB. Most glaucoma patients had been ill for less than 3 years (63.7%). Almost half of the patients reported having at least one chronic disease (49.8%). The bivariate analysis found that glaucoma patients with high school education or above (*P* < 0.05), those with a monthly income of less than 3000 RMB (*P* < 0.01), and those without other chronic diseases (*P* < 0.01) had a higher QoL. In addition, glaucoma patients complicated with cataract exerted lower scores of QoL than glaucoma patients (*P* < 0.01). Specifically, the QoL among glaucoma patients who have family history of glaucoma or cataract was significantly lower than patients without family history (*P* < 0.05).

**TABLE 1:** Demographic characteristics and clinical information of glaucoma patients and distributions of QoL.

Variables	N	%	QOL (Mean ± SD)
**Age (year)**			
≤65	89	41.4	42.19 ± 16.39
>65	126	58.6	45.02 ± 13.83
**Gender**			
Male	98	45.6	43.41 ± 15.74
Female	117	54.4	44.21 ± 14.36
**Marital status**			
Married	203	94.4	44.20 ± 15.07
Others	12	5.6	37.83 ± 12.13
**Educational level**			
Junior high school or below	128	59.6	45.67 ± 13.73
Senior high school or above	87	40.5	41.16 ± 16.35*
**Monthly income**			
<3000RMB	77	35.8	48.05 ± 15.30
3000–6000RMB	98	45.6	41.51 ± 13.40**
>6000RMB	40	18.6	41.47 ± 16.52**
**Duration of the disease**			
<3years	137	63.7	44.45 ± 14.53
≥3 years	78	36.3	42.79 ± 15.77
**With other chronic diseases**			
Yes	107	49.8	46.55 ± 15.84
No	108	50.2	41.17 ± 13.62**
**Disease types**			
glaucoma	136	63.3	46.51 ± 14.32
Glaucoma complicated with Cataract	79	36.7	39.25 ± 15.04**
**Number of operations**			
0	93	43.3	44.94 ± 14.03
1	84	39.5	43.86 ± 14.30
2	24	11.2	41.29 ± 17.26
3 or more	13	6.0	40.69 ± 21.31
**Family history of glaucoma/cataract**			
Yes	42	19.5	39.02 ± 15.33
No	173	80.5	45.02 ± 14.69*

*Significant at *P < 0.05 (two-tailed) and **P < 0.01 (two-tailed).

*Values are presented as mean ± standard deviation.

### The Correlations of Sleep Disturbance, Resilience, Quality of Life

The results in Table 2 show the correlation between sleep disturbance, resilience, and QoL. Particularly, the QoL of glaucoma patients was significantly correlated with resilience and sleep disturbance. Specifically, resilience was negatively correlated with their QoL score (*r* = −0.375, *P* < 0.01), while sleep disturbance was positively correlated with their QoL score (*r* = 0.46, *P* < 0.01). In addition, there was a negative correlation between resilience and sleep disturbance among glaucoma patients (*r* = −0.268, *P* < 0.01).

**TABLE 2:** The correlations of each continuous variable (*N* = 215).

Variable	Mean	SD	Range	1. QoL	2. Age	3. Resilience	4. Sleep disturbance
1. QoL	43.85	14.97	15∼75	1			
2. Age	66.24	12.53	30∼92	0.119	1		
3. Resilience	30.65	5.73	15∼56	−0.375**	−0.178**	1	
4. Sleep disturbance	22.33	7.99	9∼40	0.460**	−0.039	−0.268**	1

*Significant at *P < 0.05 (two-tailed) and **P < 0.01 (two-tailed).

*A higher QoL score meant the worse QoL among glaucoma patients.

### Regression Analysis of Quality of Life, Resilience and Sleep Disturbance in Glaucoma Patients

**FIGURE 1:**
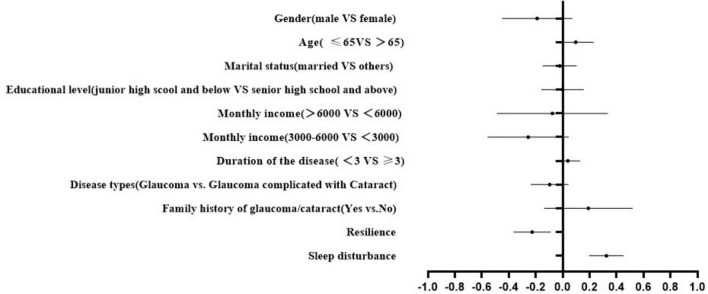
Forest graph of the hierarchical multiple regression analysis of QoL.

The forest plot (Figure 1) reflected that resilience was found to be inversely correlated with QoL scores. Conversely, sleep disturbance was positively linked with QoL scores. The final regression model explained the variance of 29.9%. The results of △R^2^ revealed that the differences in QoL explained by each variable block were 5.2, 5.1, 7.1, and 8.7%, respectively. Moreover, sleep disturbance contributed the most to the QoL among glaucoma patients. The final HMR model indicated that when sleep disturbance was added into model 4, the regression coefficient (β) between resilience and QoL increased from −0.306 to −0.226 (Table 3). The mediating role of sleep disturbance between resilience and QoL in glaucoma patients was confirmed by Sobel test (−0.306∼−0.226).

**TABLE 3:** The hierarchical multiple regression analysis of QoL (*N* = 215).

Variables	Model 1	Model 2	Model 3	Model 4
**Block 1 Demographic characteristics**				
Gender (Male vs. Female)	−0.032	−0.042	−0.072	−0.112
Age (years) (≤65 vs. >65)	−0.084*	0.145*	−0.075	0.096
Marital status (Married vs. Others)	−0.066	0.026	−0.024	0.021
Educational level (Junior high school or below vs. Senior high school or above)	−0.169*	−0.106	−0.069	−0.055
**Monthly income (RMB)**				
(>6000 vs. <3000)	−0.093	−0.071	0.001	−0.037
(3000–600 vs. <3000)	−0.169*	−0.177*	−0.127	−0.151*
**Block 2 Clinical information**				
Duration of the disease (year) (<3 vs. ≥3)		0.020	0.019	0.045
Disease types (Glaucoma vs. Glaucoma complicated with Cataract)		−0.231**	−0.199**	−0.094
Family history of glaucoma/cataract (Yes vs. No)		0.106	0.070	0.093
Block 3 Resilience			−0.306**	−0.226**
Block 4 Sleep disturbance				0.330**
R^2^	0.079	0.141	0.213	0.299
Adjusted R^2^	0.052	0.103	0.174	0.261
△R^2^	0.052	0.051	0.071	0.087

*Significant at *P < 0.05 (two-tailed) and **P < 0.01 (two-tailed).

### Mediator of Sleep Disturbance Between Resilience and Quality of Life

**FIGURE 2:**
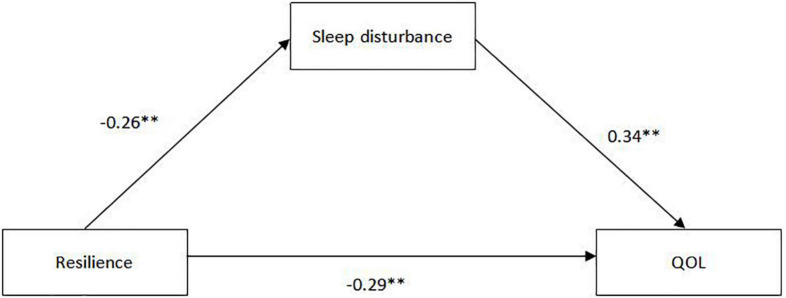
The structural equation modeling of the relationship between resilience and QoL mediated by sleep disturbance.

For the indirect effects mediated by sleep disturbance, Figure 2 demonstrates that sleep disturbance is negatively correlated with resilience (β = −0.26) and positively correlated with QoL (β = 0.34), the results were significant (*P* < 0.01), and there was a good fitting index (χ2/df <5, *P* < 0.05, GFI = 0.941, AGFI = 0.902, CFI = 0.969, TLI = 0.992, and RMSEA = 0.038). In addition, the direct impact of resilience on QoL was significant (*P* < 0.01), which was negative (β = −0.39). After adding sleep disturbance to the SEM model, the path coefficient for the association between resilience and QoL decreased significantly (β = −0.29, *P* < 0.01), Therefore, Sleep disturbance is regarded as a mediator in the model. From the results of the bias-corrected and accelerated bootstrap test (a*b = −0.1, BCa95% CI: −0.154∼−0.045), the significant mediating effect of sleep disturbance between resilience and QoL is confirmed. Therefore, we can calculate that resilience not only directly affects QoL, but also indirectly affects QoL through the mediating effect sleep disturbance.

## Discussion

There are few studies on the relationship between QoL and resilience among glaucoma patients. According to our knowledge, this study was the first attempt to examine whether sleep disturbance is a mediating factor between resilience and QoL among patients with glaucoma. Our results demonstrate that the average QoL score for patients with glaucoma was 43.85 ± 14.97, which indicates that the QoL among glaucoma patients in this study was significantly worse than the previous findings in china (28.79 ± 12.74) (31) and in Australia (30.5 ± 13.7) (32). In addition, according to our research results, the resilience of glaucoma patients (30.65 ± 5.73) was lower than that of rheumatoid arthritis patients (41.51 ± 7.07) (33). Therefore it is of upmost importance to improve the QoL for glaucoma patients.

This study revealed a significant direct correlation between resilience and QoL among patients with glaucoma, which was consistent with most previous studies. These studies showed that the QoL among patients with inflammatory bowel disease (34), recurrent coronary artery disease (35), and Parkinson’s disease (36) was positively related to their resilience. Considering that the progression of glaucoma symptoms might have a negative impact on mental health, individuals with higher level of resilience may more effectively deal with the pressure brought by having a chronic disease (37). Resilience was found to be a positive psychological factor in this study, which was particularly helpful to mitigate the negative emotions of glaucoma patients and increase their psychological adaptability (38). Compared to people with lower resilience, those who had higher resilience were able to respond to problems energetically when dealing with various pressures (such as medical expenses and disease symptoms). Accordingly, when glaucoma patients faced various symptoms including visual impairment, eye distension and headache, individuals who had higher levels of resilience were more adaptable (39), which eventually improved their QoL.

Our findings illustrate that sleep disturbance has a negative impact on the QoL of glaucoma patients, and contribute the most to the QoL among the variables included this study. Therefore, meaning that glaucoma patients with severe sleep disturbance have a lower QoL. Studies have shown that sleep disturbance has become a major risk factor for the decline of QoL (40). These studies indicate that sleep disturbance reduce the QoL among patients with fibromyalgia (41), chronic kidney disease (42), and lung cancer (43). Glaucoma patients suffer from sleep disturbance due to visual impairment and pain, such as difficulty in falling asleep, waking up early, sleep interruption, difficulty sleeping, and discomfort after waking up (44). Further, sleep disturbance causes autonomic nervous disorders and aggravates glaucoma (45), thereby reducing the Qol of glaucoma patients. These findings are consistent with current the research results (46). Hence, more attention should be paid to the measurement of intraocular pressure during sleep, among patients who are undergoing glaucoma treatment in order to adopt appropriate, and correct treatment, avoid further damage of the optic nerve and visual function, which will ultimately improve the QoL of patients with glaucoma.

Our study reveals that the effect of resilience on improving the QoL of glaucoma patients was mediated by sleep disturbance. The results show that the QoL of glaucoma patients was not only directly affected by resilience, but also indirectly affected by sleep disturbance. Glaucoma patients who reported having higher resilience were less likely to have sleep disturbance, while patients without sleep disturbance had better QoL. Studies have shown that diabetic patients with lower levels of resilience and higher levels of sleep disturbance were, more likely to suffer from depression (47), thereby affecting their QoL. Guopeng Li et al. conducted a survey with pregnant women in China and found that those with high level of resilience usually had better sleep status and were less likely to suffer from sleep disturbance (48). Consequently, their QoL could also be maintained better. Similarly, Yumei Cai et al. pointed out that resilience was negatively correlated with sleep disturbance, which was associated with positive individual health status (24). However, few studies examined sleep disturbance as a mediator between resilience and QOL among glaucoma patients. This finding may be due to: positive psychological factors alleviating the changes of individual neurohormones, resulting in improved sleep. For example, the HPA axis activation of individuals with high resilience could be maintained at an optimal level and therefore these individuals may better could cope with difficulties without excessive panic, uneasiness and depression, so as to avoid psychosomatic disorders, such as sleep disturbance, and improve their QoL (49). Another highly credible explanation is that: positive psychological factors, such as high coping self-efficacy, positive emotions, cognitive flexibility, and realistic optimism, could alleviate sleep disturbance *via* the beneficial effects on physical and mental health. Therefore, individuals with higher level of resilience could better adjust their mental state, actively treat and maintain good sleep quality when facing chronic diseases such as glaucoma, ultimately improving their QoL (50).

The findings of this study have several practical significances. Based on the empirical evidence on the positive effect of resilience on the QoL of glaucoma patients and sleep disturbance mediated in the relationship between resilience and QoL among glaucoma patients, some preliminary suggestions could be drawn. Firstly, attention and efforts of glaucoma patients to improve resilience and QoL might be diverted to promoting sleep quality rather than less easily changing factors such as disease severity (51). Secondly, it is recommended that autonomous relaxation exercises can be used by glaucoma patients, including meditation, yoga and progressive muscle relaxation, which have been proven to normalize intraocular pressure, permanently reduce psychological stress and improve resilience (52, 53). it is suggested that conduct psychological counseling for particularly serious cases in order to improve coping strategies and developed higher resilience (54).

## Limitations

Some limitations of this study should be explained. First, the clinical information collection of glaucoma is not comprehensive. There is a lack of “disease severity,” “drugs” and other clinical information. Second, this study lacks a control group, gathered data using a cross sectional survey, therefore the causal relationship between variables cannot be determined. In order to further confirm the results found in this study, there is a need for longitudinal research. Finally, the sample size of this study is limited and all of participants were undergoing outpatient treatments, which may limit the generalizability of the study.

## Conclusion

This study found that people with glaucoma have a poor QoL. Improved resilience could improve the QoL of glaucoma patients, while sleep disturbance could reduce the QoL of patients. Furthermore, sleep disturbance mediated the relationship between resilience and QoL, which could reduce the QoL among glaucoma patients. Therefore, measures should be taken to improve the QoL among glaucoma patients and strengthen their resilience training, thus promoting improved QoL among glaucoma patients.

## Data Availability Statement

The original contributions presented in this study are included in the article/supplementary material, further inquiries can be directed to the corresponding author.

## Ethics Statement

The studies involving human participants were reviewed and approved by China Medical University. The patients/participants provided their written informed consent to participate in this study.

## Author Contributions

QP contributed to acquisition and analysis of data, drafting, and revision of the manuscript. BQ was contributed to the acquisition and interpretation of data. KS was responsible for the revision of the manuscript. QC, JF, and SH were responsible for the interpretation of the data and the study design. All authors contributed to the article and approved the submitted version.

## Conflict of Interest

The authors declare that the research was conducted in the absence of any commercial or financial relationships that could be construed as a potential conflict of interest.

## Publisher’s Note

All claims expressed in this article are solely those of the authors and do not necessarily represent those of their affiliated organizations, or those of the publisher, the editors and the reviewers. Any product that may be evaluated in this article, or claim that may be made by its manufacturer, is not guaranteed or endorsed by the publisher.
